# Not Just Better Resolution: A Detailed Study of the Signal Distribution in Mid-Infrared Optical Photothermal Imaging

**DOI:** 10.1021/acs.analchem.5c03194

**Published:** 2025-09-22

**Authors:** Elisabeth Holub, Nikolaus Hondl, Sebastian Wöhrer, Bernhard Lendl, Georg Ramer

**Affiliations:** † Institute of Chemical Technologies and Analytics, 27259 TU Wien, 1060 Wien, Austria; ‡ Christian Doppler Laboratory for Advanced Mid-Infrared Laser Spectroscopy in (Bio-)process Analytics, 27259 TU Wien, 1060 Wien, Austria

## Abstract

The spatial resolution of an imaging system is generally evaluated by analysis of the point spread function (PSF), that is, the system response to a point source in the object plane. The PSF, albeit a fundamental concept in light microscopy, has been sparsely studied in optical photothermal infrared (O-PTIR) imaging. Although there is only a minor difference between the photothermal and optical intensity distributions in the radial direction, the axial photothermal signal is markedly different from the imaging response of conventional light microscopes. Previous studies of particles beyond the visible diffraction limit have shown that the O-PTIR signal has two lobes and strongly varies with the offset between the foci of the pump and the probe laser. We will therefore present theoretical and experimental data to investigate the radial and axial intensity distributions of a commercial Raman microscope and a custom O-PTIR transmission instrument, with particular emphasis on the axial PSF of the O-PTIR system. Our data suggest that the two-lobed signal shape is also present in objects on the order of the probe beam wavelength.

## Graphical abstract

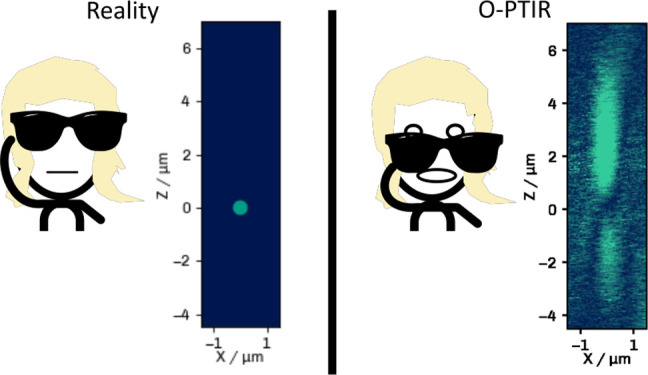

## Introduction

Optical photothermal infrared (O-PTIR) spectroscopy is a nondestructive, label-free pump–probe technique that exploits the local refractive index change in the sample upon absorption of infrared (IR) radiation. This refractive index perturbation is probed by a second laser, whose wavelength is chosen to be in the visible spectral range. Using a visible probe instead of an infrared beam, O-PTIR spectroscopy can enhance resolution beyond the limits achieved in conventional IR spectroscopy while still offering high chemical specificity.^1–4^ The field has seen a myriad of applications ranging from nanoparticles^5–8^ to formalin-fixed biological samples,^9^ live cells,^2^ and even organisms.^10,11^ However, the theoretical understanding of the signal mechanism has not kept pace with the growth in applications. Hence, to better understand the imaging capabilities of O-PTIR spectroscopy, we are striving here to determine the imaging response for the technique.

In marketing material and academic work alike, O-PTIR is often called “stainless staining,”^12^ suggesting the technique works similarly to conventional label-based microscopic imaging techniques, such as confocal fluorescence. In contrast to traditional staining, where different dyes (e.g., hematoxylin and eosin—H&E) are used to visualize and quantify the presence of a specific compound and improve histological analysis, O-PTIR can provide chemical information without the use of dyes.

The thermal lensing effect exploited in photothermal microscopy is based on a change in temperature, which is associated with a change in the optical path length.^13^ In traditional thermal-lens spectroscopy, two types of experimental setup are used, mode-matched and mode-mismatched.^14^ In the mode-matched configuration, both the pump and the probe beam are focused by using one and the same lens to achieve similar Rayleigh ranges and spot sizes. For this reason, both beams commonly have wavelengths in the visible spectral range.

Mode-mismatching^15,16^ has been shown to increase sensitivity,^14^ which require a high spatial resolution and compact design.

In the present work, we aim to describe the intensity distribution around isolated spherical or ellipsoidal objects of several hundreds of nanometers to a few micrometers in diameter using focused Gaussian beams and pump laser wavelengths in the mid-IR region. This description is particularly important for imaging applications, where the detection volume is only a few cubic micrometers.

The capability of a conventional microscope to resolve sample features is described by the three-dimensional point spread function (PSF), that is, the system’s response to a point object in focus. In a diffraction-limited optical instrument, even an ideal point will be subject to blurring owing to diffraction and aberration effects. The resulting image intensity distribution is therefore a measure of the resolution of the microscope.^17,18^

While both experimental and theoretical PSFs have been extensively explored for light microscopy, theoretical and practical research on the performance of photothermal instruments is rather scarce. Since O-PTIR instruments were first developed for surface imaging in reflection mode, experimental studies have focused primarily on lateral spatial resolution.^3,4^ Similarly, O-PTIR detection of nanoparticles is mainly concerned with the achievable signal-to-noise ratio (SNR)^5,6^ and detection limits^19^ rather than spatial resolution, which is attributable to the fact that nanoparticles are beyond the expected visible resolution limit. However, the most important reason for the lack of a systematic approach lies in the complexity of the photothermal signal response, which is based on the interaction between the probe and the pump beam and is hence not a point-spread function in the conventional sense.

In the radial plane, there is only a minor difference between photothermal and optical imaging resolution as long as the steady-state regime applies, that is, when the thermal diffusion length *R*_th_ is larger than the spot size (see “Point Spread Function in O-PTIR Microscopy” section). In this case, resolution is governed solely by the wavelength of the probe beam^20^ and the numerical aperture (NA) of the focusing and collection objectives.

In contrast, the axial photothermal signal is intrinsically different from the intensity distribution of conventional light microscopes. Transmission experiments have shown that the axial photothermal signal has two lobes^21,22^ and depends on the offset Δ*z* between the waist positions of the lasers. However, no systematic description of the phenomenon was given.

Several theoretical models have been evaluated,^7,23,24^ but so far their practical application has been confined to nanoparticles beyond the visible diffraction limit. Moreover, nanoscale pump–probe experiments use probe and pump wavelengths in the visible or near-infrared range, that is, with similar wavelengths. Conversely, in O-PTIR, the wavelengths of the mid-IR pump beam and the visible probe beam are roughly 1 order of magnitude apart. In this work, we show that the diffraction model developed by Selmke et al.^23,24^ (“nanolens model”) still holds for objects on the order of the wavelength of the probe beam and pump laser wavelengths in the mid-IR range.

The present work evaluates the imaging response of a custom O-PTIR transmission instrument, which was developed with the aim of providing a wide application range, including imaging in aqueous environments. The radial and axial PSFs of the custom O-PTIR instrument are compared to a state-of-the-art Raman microscope through experiments and theoretical description of the signal.

Confocal Raman microscopy is a well-established chemical imaging technique that offers both high specificity and visible-range resolution. Raman instruments rely on the inelastic scattering of a monochromatic light source, which is focused on a small region of the sample. The scattered light is collected and focused on a pinhole.^25^ Raman was chosen here for comparison as it probes similar sample information as O-PTIR - molecular vibrations - using visible wavelength light but, in contrast to O-PTIR, has a purely optical signal generation without photothermal contributions.

Typically, Raman instruments are designed in the reflection mode. Although a versatile tool, Raman spectroscopy suffers from a very low cross section, and the weak signal may be masked by autofluorescence of the sample.^26^ O-PTIR, on the other hand, does not suffer from autofluorescence and can, in fact, even leverage autofluorescence.^27^

We will present theoretical calculations and experimental data to compare the radial and axial intensity distributions of both instruments with particular emphasis on the axial PSF of the O-PTIR system. Finally, we will discuss the ramifications of the axial distribution of the photothermal signal for O-PTIR imaging and spectroscopy.

## Theoretical Background

### Point Spread Function in Optical Microscopy

For bright-field microscopy, the collected light is at least in part coherent, and the intensity of an image is given by the three-dimensional convolution 
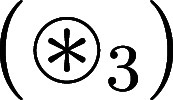
 of the amplitude transmittance (reflectance) *o*(*r*, *z*, λ) of the object and the point spread function,^17,25^



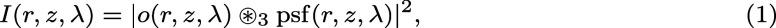

1
where λ is the wavelength, *z* is the axial coordinate, and $r=\sqrt{x^{2}+y^{2}}$ is the radial coordinate.

The PSF of a confocal transmission microscope reflects the fact that the light passes through two objectives that might have different specifications:

$$\mathrm{psf}(r,z,\lambda )={\mathrm{psf}}_{\mathrm{ill}}(r,z,\lambda ){\mathrm{psf}}_{\mathrm{col}}(r,z,\lambda )$$ 2

where psf_ill_ and psf_col_ are the (nonparaxial) intensity distributions of the illumination and collection arm. For a confocal reflection setup, psf_ill_ = psf_col_.

Even though the incident laser light is linearly polarized (here, the x direction has been selected as the direction of polarization), the electric field in the image plane has components in all three directions *x*, *y*, and *z*, with complex vector components *e*_*i*_:^28,29^

$$\vec{E}(r,z,\lambda ,\theta )=\frac{i\pi }{\lambda }([e_{0}+e_{2}\cos\;2\theta ]\vec{x}+[e_{2}\sin\;2\theta ]\vec{y}+[2i\cdot e_{1}\cos\;\theta ]\vec{z})$$ 3

where θ represents the azimuth angle, *z* is the axial coordinate, and *r* is the radial coordinate in the image plane.

Imaging requires the focusing of light by optical elements. In the simplified case of an aplanatic, that is, aberration-free, optical system, the wavefronts are spherical. The vectorial components in the image plane are therefore conveniently expressed in spherical polar coordinates (*r*, θ′, ϕ). The components *e*_0_ = *e*_0_(*k*, *r*, *z*, α), *e*_1_ = *e*_1_(*k*, *r*, *z*, α), and *e*_2_ = *e*_2_(*k*, *r*, *z*, α) are^29^

$$\begin{array}{l} e_{0}=\int_{0}^{\alpha }P(\theta^{\prime })(1+\cos\;\theta^{\prime })J_{0}(kr\sin\;\theta^{\prime })\exp(-ikz\cos\;\theta^{\prime })\sin\;\theta^{\prime }d\theta^{\prime },\\ e_{1}=\int_{0}^{\alpha }P(\theta^{\prime })J_{1}(kr\sin\;\theta^{\prime })\exp(-ikz\cos\;\theta^{\prime })\sin^{2}\;\theta^{\prime }d\theta^{\prime },\\ e_{2}=\int_{0}^{\alpha }P(\theta^{\prime })(1-\cos\;\theta^{\prime })J_{2}(kr\sin\;\theta^{\prime })\exp(-ikz\cos\;\theta^{\prime })\sin\;\theta^{\prime }d\theta^{\prime }, \end{array}$$ 4

where *J*_0_, *J*_1_, and *J*_2_ denote the Bessel functions of the first kind of order 0, 1, and 2, *P*(θ′) is the apodization function of the objective, θ′ is the aperture angle, α is the maximum aperture angle and *k* = 2π*n*/λ. Because *k* depends on the wavelength λ, so does $\vec{E}$ .

Using the Abbe sine condition, *P*(θ′) can be simplified by

$$P(\theta^{\prime })=P(r)\sqrt{\cos\;\theta^{\prime }}$$ 5

with *P*(*r*) being the amplitude transmittance at the objective aperture. For a uniform circular aperture, *P*(*r*) = 1.^17^

Equations 3 and 4 also show that strictly speaking the field is not symmetric around the axis of beam propagation. However, for small angles (i.e., a sufficiently low NA), the Bessel functions *J*_1_ and *J*_2_ are small compared to those of *J*_0_. As a consequence, the vectorial components *e*_1_(*J*_1_) and *e*_2_(*J*_2_) are negligible, and only *e*_0_(*J*_0_) contributes to the field:

$$\vec{E}(r,z,\lambda ,\theta )\approx \vec{E}(r,z,\lambda )=\frac{i\pi }{\lambda }e_{0}\vec{x}$$ 6

This eliminates the dependence on angle θ, which in turn implies that the field in the image plane is radially symmetric around the direction of propagation (*z*). Hence, the point spread function, which is calculated from the electric field, does not differ between the *x* and *y* directions if the NA is small. As exp­(−*ikz*cosθ) = 1 for *z* = 0, the integral is real in the focal plane. Outside of the focal plane, the amplitude of the electric field is complex.

Because the PSF is given by the field intensity distribution and intensity is proportional to the squared modulus of the electric field, it follows that

$$I(r,z,\lambda )\propto \vec{E}(r,z,\lambda ){\vec{E}}^{*}(r,z,\lambda )=|E(r,z,\lambda ){|}^{2}\to I(r,z,\lambda )\propto |e_{0}{|}^{2}$$ 7

Consequently, the PSF of a confocal microscope is proportional to

$$I(r,z,\lambda ,\theta )\propto |e_{0,ill}{|}^{2}\cdot |e_{0,\mathrm{col}}{|}^{2}$$ 8

where *e*_0,ill_ = *e*_0_(*k*, *r*, *z*, α_ill_) and *e*_0,*col*_ = *e*_0_(*k*, *r*, *z*, α_col_).

### Point Spread Function in O-PTIR Microscopy

In contrast to optical microscopy, photothermal microscopy harnesses the local refractive index change (“thermal lens”) induced in a sample by a heating laser beam (Figure 1). The photothermal transmission signal is the change in transmitted probe intensity due to this thermal lensing effect.^23,30^ The relative signal is then^23,24^

$$\Phi (r,z)=\frac{|E(r,z){|}_{\Delta n(\Delta T)}^{2}-|E(r,z){|}_{\Delta n=0}^{2}}{|E(r=0,z){|}_{\Delta n=0}^{2}}$$ 9

**Figure 1:**
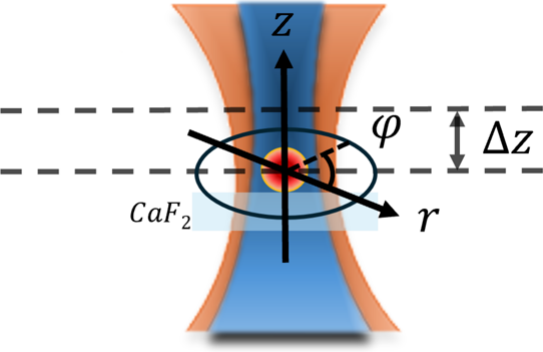
Principle of O-PTIR microscopy. A spherical particle (red circle) absorbs a pump beam (orange) and changes the refractive index in its surroundings (yellow ring around the particle). The refractive index change in the sample is detected by a visible probe beam (blue). The laser foci are offset by the axial distance Δ*z*. Here, the particle center is in the focus of the probe beam at (*z* = 0, *r* = 0).

When the thermal diffusion length $R_{\mathrm{th}}=\sqrt{\frac{2\kappa }{C_{p}\rho \Omega }}$ ^5,6,31^ (where *C*_*p*_ is the specific heat capacity, ρ is the density and Ω is the angular modulation frequency of the pump laser) is larger than the spot size radius of the probe beam, a steady-state approximation can be made.^5,24,32^ In this steady-state regime, the temperature change introduced by a point heat source in an infinite medium is,^33^ p. 261

$$\Delta T\left(r\right)=\frac{P_{diss}}{4\pi \kappa r}$$ 10

where *P*_diss_ = σ_abs_ *I*_0,*h*_ is the dissipated heat.

Using linear approximation, the refractive index perturbation induced in the vicinity of a small particle after the absorption of the pump laser can be described by^23,30^

$$n(r)\approx n(T_{0})+\frac{dn}{dT}\Delta T(r)=n_{0}+\frac{dn}{dT}\frac{P}{4\pi \kappa r}=n_{0}+\Delta n\frac{R}{r}$$ 11

where *T*_0_ is the initial temperature, *n*_0_ is the unperturbed refractive index of the surrounding medium, $\frac{dn}{dT}$ is the thermo-optic coefficient of the medium at the probe beam wavelength, Δ*T* is the temperature change induced by the pump laser, Δ*n* is the corresponding change in the refractive index, and *R* is the radius of the particle.

The above equation represents an ideal thermal lens in an isotropic medium resulting from a steady-state temperature profile, where the refractive index change at the position *z* reads as^23,30^ (see S-2, (S1)-(S2))­

$$\Delta n(z,\Delta z)=\frac{\sigma_{\mathrm{abs}}I_{0,h}}{4\pi \kappa R}\frac{dn}{dT}{\left(1+\frac{(z-\Delta z{)}^{2}}{z_{R,h}^{2}}\right)}^{-1}$$ 12

with σ_abs_ denoting the absorption cross section of the particle, κ being the thermal conductivity of the ambient medium, Δ*z* being the offset between the pump and the probe beam waist, $I_{0,h}=\frac{2P_{\max,h}}{\pi \omega_{0,h}^{2}}$ representing the heating beam peak intensity at the sample, and $z_{R,h}=\frac{\pi \omega_{0,h}^{2}}{\lambda_{h}}$ being the Rayleigh range of the heating beam at wavelength λ_*h*_ and beam waist ω_0,*h*_.

The peak intensity of the pump beam *I*_0,*h*_ is related to the peak power of the IR laser *P*_max_, which can be deduced from the average power *P*_avg_ and duty cycle β via *P*_max_ = *P*_avg_/β (see S-2, (S3)).

The following description is based on the Fresnel diffraction formalism.^34,35^ The diffraction by apertures can be used to study the effect of a pinhole on a beam of light. Similarly to the transmission of light through an opening, one can define an inverse aperture, blocking the central part of the light. It has been shown that the axial electric field behind a sphere can be very well approximated by the field of a disk in the far field.^36^ For this reason, the diffraction of a laser beam by a spherical object (e.g., a polymer bead) can be approximated by an inverse circular aperture.^23^

In a very general form, an on-axis Gaussian beam field *E*_0_ propagating in the z direction is transformed by a circular aperture as follows:^37^

$$E(x,y,z=0)=T(x,y)E_{0}(x,y,z=0)$$ 13

where *x* and *y* are the lateral coordinates in the aperture plane and *T*(*x*, *y*) is the applicable aperture transmission function.

After transformation to spherical coordinates and partial integration, eq 13 can be expressed as^23,24^

$$\begin{array}{l} E(r,z)=\frac{k}{iz}\exp\left(i\frac{kr^{2}}{2z}+ikz\right)\cdot \\ \int_{R}^{\infty }T(\rho )U_{a}(\rho )\exp\left(\frac{ik\rho^{2}}{2z}\right)J_{0}\left(\frac{k\rho r}{z}\right)\exp(-i\Delta \chi (\rho ))\rho d\rho , \end{array}$$ 14

where ρ is the radial coordinate of the aperture plane, *r* is the radial coordinate of the image plane, *J*_0_ is the zeroth-order Bessel function, *U*_*a*_ is amplitude of the probe beam, Δχ­(ρ) ≈ const. – 2*k*_0_ *R*Δ*n*lnρ is the phase advance and *k* = *k*_0_ *n*_0_ is the wavenumber.

Assuming a Gaussian probing field (S7), one can express the relative photothermal signal (eq 9) at a specific detection angle θ as^23,24^

Φ(θ,z)=exp(−k2tan2θRe(ζ−1)2)(exp(2RΔnk0arg(ζ))·|Γ(1+iRΔnk0)F11(−iRΔnk0,1,k2tan2θ4ζ)|2−1) 15

In the above equation, the curvature term ζ for large distances from the focus is approximately $\zeta (z)\approx \frac{1}{\omega^{2}(z)}+\frac{ik}{2R_{c}(z)}$ ,^23^ with *R*_*c*_ denoting the radius of curvature of the probe beam (see S-4), Γ is the gamma function, and _1_ *F*_1_ is the confluent hypergeometric function of the first kind.

Because the signal is collected at all angles within the cone defined by the collection objective, eq 15 must be integrated over the domain defined by the collection angles θ_min_ and θ_max_, with $\theta_{\max}=\sin^{-1}\left(\frac{NA}{n_{0}}\right)$ and θ_min_ = 0 for objectives without a central beam stop. Finally, one obtains the detected relative photothermal signal

$$\Phi_{tot}(\theta_{\min},\theta_{\max},z)=A\times 2\pi \int_{\theta_{\min}}^{\theta_{\max}}\Phi (\theta ,z)\frac{\sin\;\theta }{\cos^{3}\;\theta }d\theta$$ 16

with the normalization factor

$$A=\frac{2z_{R}^{2}}{\pi w_{0}^{2}}{\left(\exp\left(-\frac{2\tan^{2}\theta_{\min}z_{R}^{2}}{w_{0}^{2}}\right)-\exp\left(-\frac{2\tan^{2}\theta_{\max}z_{R}^{2}}{w_{0}^{2}}\right)\right)}^{-1}$$ 17

As the Gouy phase (see S-4) does not depend on ρ, it simply cancels out in the intensity quotient. The relative signal is therefore independent of the Gouy phase. Furthermore, the relative representation of the signal does not reflect changes in the probe power.

## Materials and Methods

### Sample Preparation

Polystyrene (PS) beads with a diameter of 500 nm (L3280, Sigma) were diluted with deionized water and dried on CaF_2_ disks (Crystran Ltd.) of 1 mm thickness at ambient temperature (294 K). The CaF_2_ substrates had been cleaned with a detergent (Alconox, Inc.) prior to use.

### Instruments and Settings

#### O-PTIR Instrument

The photothermal images were recorded with a custom transmission setup (Figure 2). Two laser sources, a tunable EC-QCL (MIRcat-QT-z, Daylight Solutions) and a visible CW diode laser (LBX-633, Oxxius), are arranged in a confocal geometry.

**Figure 2:**
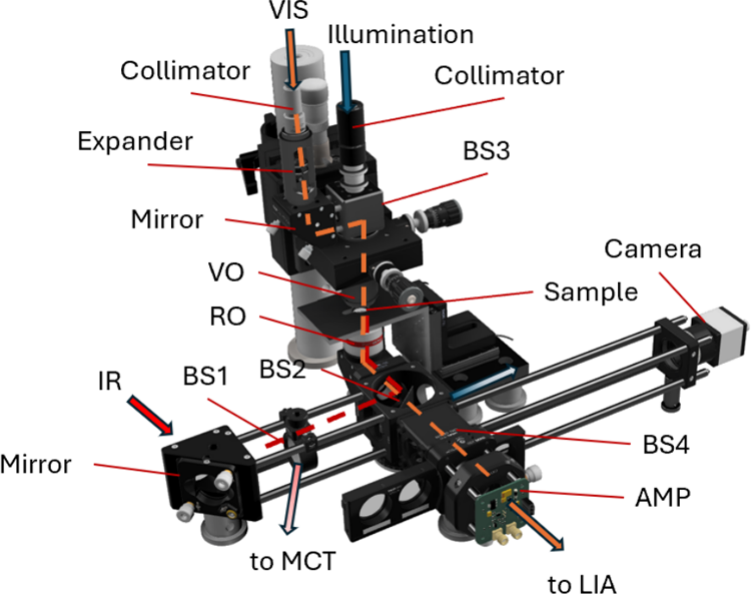
Custom O-PTIR setup. IR: infrared laser; VIS: visible laser; VO: visible light objective; RO: reflective objective; AMP: preamplifier; BS: beam splitter; LIA: lock-in amplifier. IR beam path: red dashed line; visible beam path: orange dashed line.

The visible laser has a fixed wavelength of 633 nm; after collimation and expansion, it is focused on the sample by a 0.5 NA visible light objective (VO, Olympus LMPLFLN, 50×, WD = 10.6 mm). The visible probe power is approximately 0.5 mW at the sample. This was the lowest power setting at which the signal quality was still sufficient for imaging. Minimizing laser power is crucial to avoid damage to the sample and heating by the probe laser.

A wedged CaF_2_ window (BS1) directs part of the IR laser power toward a mercury cadmium telluride detector (MCT). The MCT power readout serves as a reference for power normalization in spectra.

A reflective objective (RO, Thorlabs LMM40X-P0, 40×, WD = 7.8 mm) with NA = 0.5 is used to focus the IR beam to the back of the sample and to collect the visible laser after transmission through the sample. The reflective objective comprises two mirrors, one of which obscures the central part of the beam, making the RO an annular aperture that can be harnessed to monitor the photothermal lensing effect. A change in the refractive index of the sample will modify the diameter of the transmitted beam at the annular aperture, and thus change the fraction of beam power transmitted through the aperture, which will translate to a change in the detected power.

A beam splitter (BS4) passes the transmitted visible laser light on to the detector but redirects all other light to a camera.

The photothermal signal is detected by a custom preamplifier (AMP) comprising a photodiode and then filtered by a lock-in amplifier (LIA, MFLI 500 kHz, Zurich Instruments). The LIA demodulates the photodiode signal at the laser repetition rate and outputs the amplitude via an API. For imaging, the amplitude is recorded as a function of the sample position. For recording spectra, the infrared laser is tuned at a constant tuning speed of 100 cm^–1^ s^–1^, and the signal amplitude is recorded as a function of the pump beam wavelength.

The modulation frequency and duty cycle of the tunable pump laser were set to 50 kHz and 0.025, respectively. At these settings, the thermal diffusion length *R*_th_ is ≈12 μm, guaranteeing compliance with the steady-state assumption (see “Point Spread Function in O-PTIR Microscopy” section). Before imaging, a spectrum was recorded to identify suitable wavenumber settings (see “Data Processing and Input Parameters” section). The IR laser path was flushed with dry air to keep humidity low (<2%).

The pixel size can be regulated via the step size of high-precision slip-stick piezo stages (ECS 5050, attocube). A pixel size of 50 nm × 50 nm was selected for the *xy* plane and 50 nm × 200 nm for the *xz* plane.

#### Raman Microscope

The WITec alpha300 RSA (WITec GmbH, Ulm, Germany) is a confocal Raman microscope that is equipped with several objectives and lasers in the visible and NIR ranges. For the best comparability with the custom O-PTIR system, the 0.55 NA objective (Nikon CF IC Plan ELWD) was selected. By the same token, the 633 nm HeNe laser was used for all Raman measurements. The laser power was set to 5 mW.

The sample is moved by a piezoactuated flexure stage. Again, the pixel size is determined by the step size, which was set to 50 nm in the *xy* and 200 nm in the *z* direction. The integration time was 5 s.

### Data Processing and Input Parameters

#### Spectra and Preprocessing

To extract the photothermal signal, the in-phase and out-of-phase signals were recovered from the lock-in data and normalized against the transmission signal for every pixel. The selection of wavenumber settings for the EC-QCL was based on band maxima determined from a typical polystyrene spectrum (Figure 3a). A set of three wavenumbers was identified: the CH_2_ bend at 1450 cm^–1^ ^19^ and the aromatic C=C stretching bands at 1490 and 1602 cm^–1^.^38^

**Figure 3:**
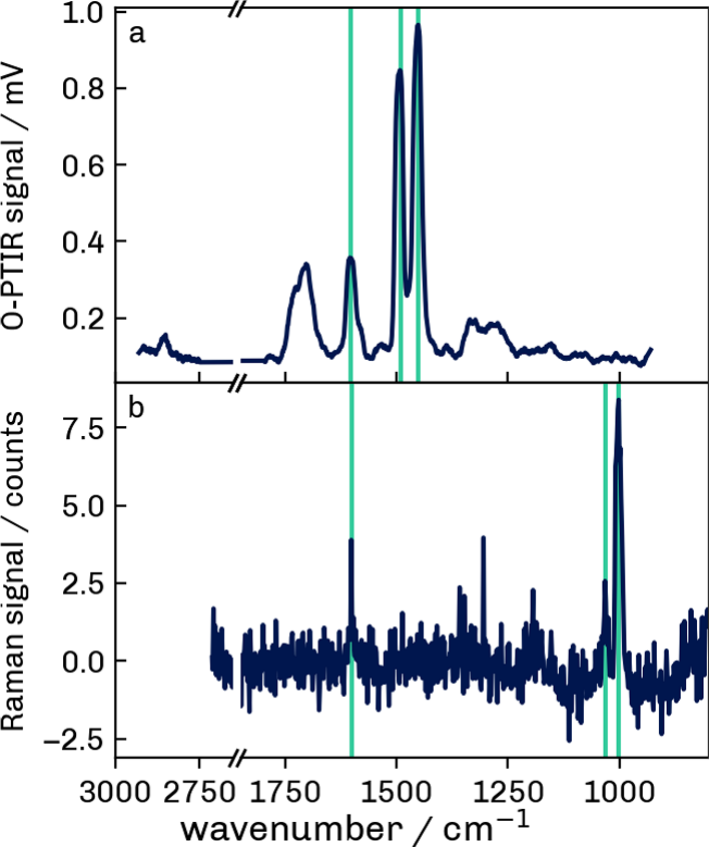
Typical (a) O-PTIR and (b) Raman spectra of a PS bead. The O-PTIR spectrum was smoothed using a Savitzky-Golay filter. The green lines mark characteristic wavenumbers: (a) 1450, 1490, and 1602 cm^–1^; (b) 1003, 1032, and 1602 cm^–1^.

The Raman graphs and spectra were extracted from the WITec file format with a Python wrapper. While photothermal images were recorded at a few select wavenumbers only, the Raman instrument is designed to collect hyperspectral data. The Raman image contrast was then obtained by integrating over the band between 980 and 1014 cm^–1^ (PS ring breathing mode^39^) as verified from the spectra. The reason for the different band positions in Raman and O-PTIR spectroscopies lies in the physical principles underlying the two techniques. While the O-PTIR bands arise from the absorption of infrared radiation, Raman spectroscopy is based on the inelastic scattering of visible light. A typical Raman spectrum is presented in Figure 3b.

All further data processing was carried out in Python 3.11.6. For both instruments, the *xz* images were rotated to correct for misalignment and sample tilt (15° for Raman and 2.5–4° for O-PTIR). These rotation angles were determined by searching for the rotation leading to the highest intensity along the vertical axis. PSFs were calculated by taking the mean of three central lines in the direction of interest in each bead image. The intensity distribution in the image plane of a microscope (see “Point Spread Function in Optical Microscopy” section) can be well approximated by a Gaussian. For this reason, a Gaussian function was fitted to the Raman data in *xy* and *xz* as well as to the *xy* photothermal signal distribution. The resolution was estimated via the fwhm of the resulting fit.

#### Parameters Used for Theoretical Calculations

To mathematically describe the experimental axial photothermal PSF, the normalized signal was fitted to ([Disp-formula eq16]) using the actual system parameters (Table 1). The beam waists were derived from the theoretical radial PSFs for use of the visible beam and the visible objective as well as the IR beam and the reflective objective. The variables to be fitted were the focus offset Δ*z* and the absorption cross section σ of PS at 1450 cm^–1^.

**Table 1:** Input Parameters for Calculation of the Axial O-PTIR Signal

parameter	symbol	value	unit
VIS beam waist radius	ω_0,*p*_	0.53	μm
VIS beam wavelength	λ	0.633	μm
IR beam waist radius	ω_0,*h*_	5.0	μm
IR beam wavelength	λ_*h*_	6.895	μm
bead radius	*R*	0.25	μm
min angle RO	θ_min_	21	°
max angle RO	θ_max_	30	°
thermo-optic coefficient @633 nm	$\frac{dn}{dT}$	–9e-7 ^40^	K^–1^
refractive index air	*n*_0_	1.0003 ^41^	
thermal conductivity air	κ	0.0262 ^42^	W/Km
avg. IR power @ 1450 cm^–1^	*P*_avg_	6.6	mW
EC-QCL duty cycle	β	0.025	
EC-QCL modulation frequency	Ω	50	kHz

## Results and Discussion

### Theoretical PSFs

The theoretical radial and axial PSFs for the Raman microscope follow from eqs 4 and 6, with the fwhm values indicating the resolution of the imaging system. In the radial direction, a similar PSF can be obtained for the O-PTIR instrument. Because features at the subnanometer scale cannot be resolved by optical systems, the discrepancy between the HeNe wavelength (632.8 nm) and the diode laser (633 nm) was neglected, and all calculations were performed for a wavelength of 633 nm.

Figure 4 displays the radial (a) and axial (b) PSFs for the confocal Raman microscope with a single objective and the corresponding PSF for the specifications of the confocal O-PTIR instrument. One should note that the collecting objective of the O-PTIR setup has a central beam stop, which increases θ_min_ to approximately 21°.^8^

**Figure 4:**
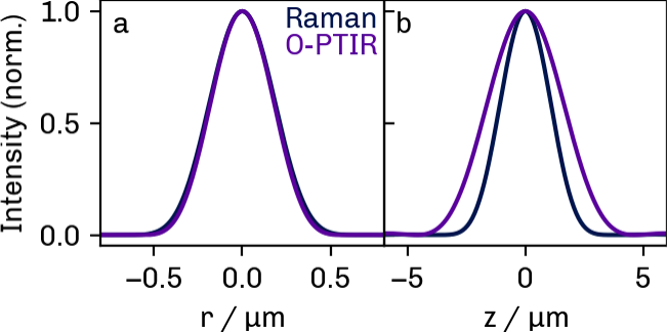
Radial (a) and axial (b) PSFs of an optical microscope with the specifications of the Raman (black) and O-PTIR (red) instruments. The Raman microscope uses a single 0.55 NA objective; the confocal O-PTIR setup has two 0.5 NA objectives, one of which has a Schwarzschild design.

For the Raman microscope, the theoretical fwhm at a laser wavelength of 633 nm is 420 nm in the *xy* plane and 2.4 μm in the axial direction. With its similar specifications, the O-PTIR instrument has a theoretical radial resolution of 460 nm. The axial fwhm for a confocal microscope with the same specifications as the custom instrument is 3 μm. The higher resolution limit for the O-PTIR configuration as compared to that of the Raman instrument is due to the use of a reflective objective and slightly lower NA.

In contrast to the axial PSF of a light microscope, the axial photothermal signal does not necessarily have a single central maximum. Inserting the values of Table 1 into eqs 16 and 15 and varying the offset between the foci of the two lasers, one finds that the photothermal signal is two-lobed for axial focus offsets up to ≈15 μm. This situation is illustrated in Figure 5a. The two signal lobes are symmetric when there is a perfect axial overlap (Δ*z* = 0) between the beam waists of the IR and the visible laser. The second lobe becomes less pronounced with an increasing axial distance between the laser foci and eventually disappears into the noise floor.

**Figure 5:**
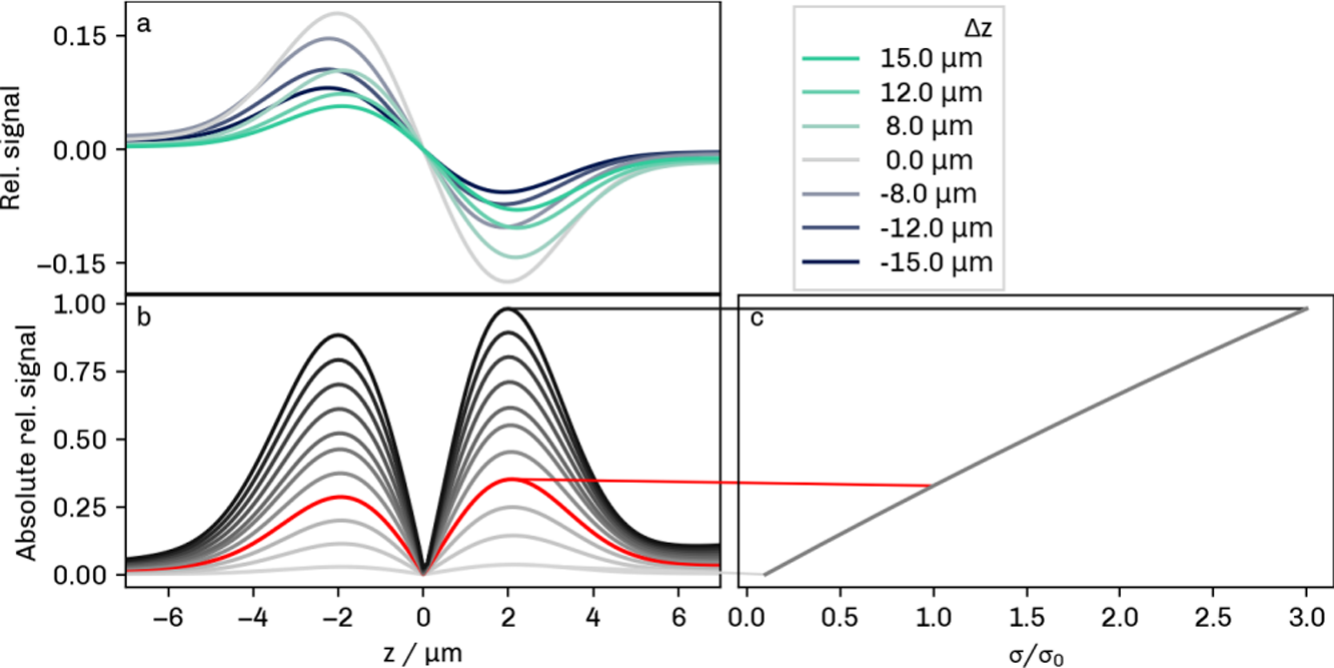
Theoretical behavior of the axial O-PTIR signal at different focus offsets Δ*z* (a) and absorption cross sections (b). The red line in (b) indicates the signal at an expected absorption cross section of σ_0_ = 0.065 μm^2^. The bead center is at position *z* = 0. Linear dependence on the absorption cross section is illustrated in (c). Settings: θ_min_ = 15°, ω_0.*h*_ = 4.6 μm, ω_0.*p*_ = 0.7 μm.

Furthermore, it should be emphasized that the position of the maximum signal does not coincide with the center of the bead and shifts with the focus offset. The signal intensity decreases considerably with large focus offsets of several micrometers.

Apart from the laser focus offset, the photothermal signal strongly depends on the absorption cross section of the scattering object. Figure 5b illustrates the signal calculated for various absorption cross sections σ with the parameters of a transmission O-PTIR instrument using a reflective objective (θ_min_ > 0). The red graph in (b) shows the signal obtained for an estimated absorption cross section of σ_0_ = 0.065 μm^2^ at 1450 cm^–1^. Please note that Figure 5b shows the absolute signal, as recovered from the experimental O-PTIR image. In fact, the maximum relative signal scales linearly with the absorption cross section (Figure 5c).

### Experimental PSF of the Raman Microscope

The Raman image obtained in the *xy* plane is presented in Figure 6a. The PSFs for the *x* and *y* directions were calculated from the mean of three central lines (central lines indicated in red). The data points representing the normalized intensity along each line and the corresponding Gaussian fits are shown in Figure 6b,c. The fits (red lines) established a fwhm value of 738 ± 30 nm (standard deviation) in *x* and 840 ± 49 nm in *y* direction.

**Figure 6:**
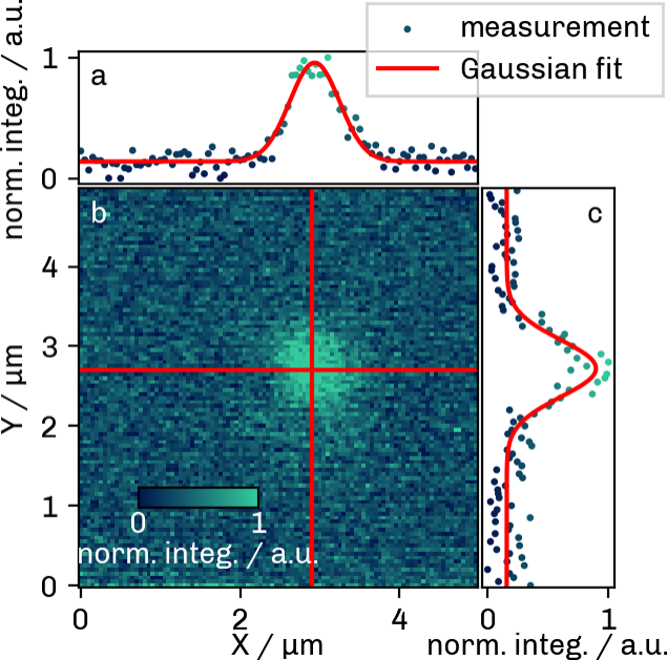
Raman *xy* image (b) of a PS bead and resulting PSFs along the red lines in *x* (a) and *y* (c) directions.

Similarly, Figure 7 illustrates the *z*-scan performed with the Raman microscope and the axial resolution derived therefrom. The image was rotated by 15° prior to PSF evaluation. The fwhm of the corresponding fit (Figure 7b, red line) was 4.377 ± 0.216 μm. The variation in image contrast, as manifested by lighter and darker areas, can be explained by changes in ambient light conditions in the laboratory during long-term measurement. The ambient light was minimized by using an intransparent cover around the sample to reduce the effects of stray light.

**Figure 7:**
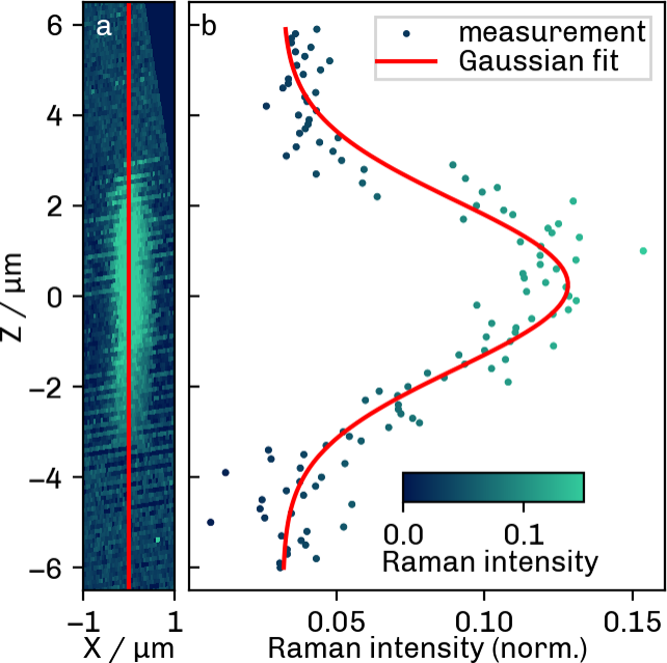
Axial Raman scan (a) and resulting PSF (b) along the red line.

### Experimental PSF of the O-PTIR Microscope

Bearing in mind that the photothermal signal depends on both the probe and the pump laser, O-PTIR images of 500 nm beads were recorded at several IR wavenumbers for validation (see “Data Processing and Input Parameters” section).

To exclude beam ellipticity as a reason for beam asymmetries, the visible diode laser beam was examined just before the objective lens by using a pyroelectric laser beam profiler (Pyrocam III, Spiricon). The evaluation of the beam width was performed in the dedicated software “BeamGage”, which relies on the second moment calculation for a TEM00 Gaussian beam. The beam width *D*_*x*,*y*_ was found to be 1.049 mm in *x* and 1.059 mm in *y* direction, resulting in an ellipticity ϵ of $\epsilon =\frac{D_{x}}{D_{y}}=0.99$ . Considering that ϵ = 1 constitutes a perfectly circular beam, the difference in beam width can be considered negligible in the present case.

#### Radial PSF

Figure 8 illustrates the photothermal image obtained in the radial plane at an IR wavelength of 1450 cm^–1^. A second image was recorded at 1490 cm^–1^ (see Section S-5, Figure S3). The fwhm was obtained from Gaussian fits along the mean of three central lines (see “Experimental PSF of the Raman microscope” section). The resulting fwhm’s and the one standard deviation errors were Δ*x* = 653 ± 29 nm and Δ*y* = 668 ± 44 nm for the 1450 cm^–1^ image, and Δ*x* = 615 ± 26 nm and Δ*y* = 613 ± 32 nm for the 1490 cm^–1^ image.

**Figure 8:**
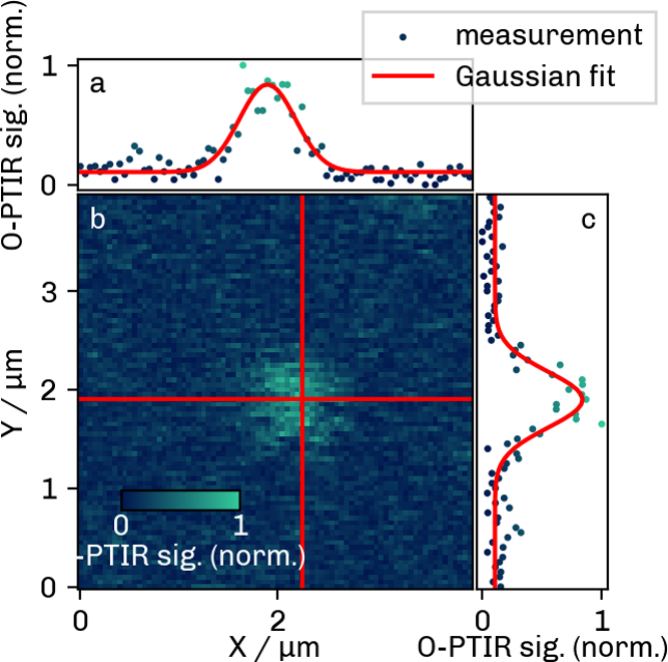
O-PTIR *xy* image at 1450 cm^–1^(b) and resulting PSFs in *x* (a) and *y* (c) directions.

The discrepancies in the experimental spatial resolution at the two wavenumber settings are largely attributed to differences in the noise dynamics, which will have an effect on the Gaussian fit and thus on the resolution defined as the fwhm value. Overall, the data corroborate the assumption that spatial resolution in photothermal imaging is predominantly determined by the wavelength of the probe laser^1^ and independent of the wavelength of the pump laser.

#### Axial PSF

Figure 9 shows the photothermal images recorded in the *xz* plane at 1450 cm^–1^ The resulting line scan (b) represents the axial PSF of the photothermal instrument, which can be explained by the theoretical model of Selmke et al.^7,23^

**Figure 9:**
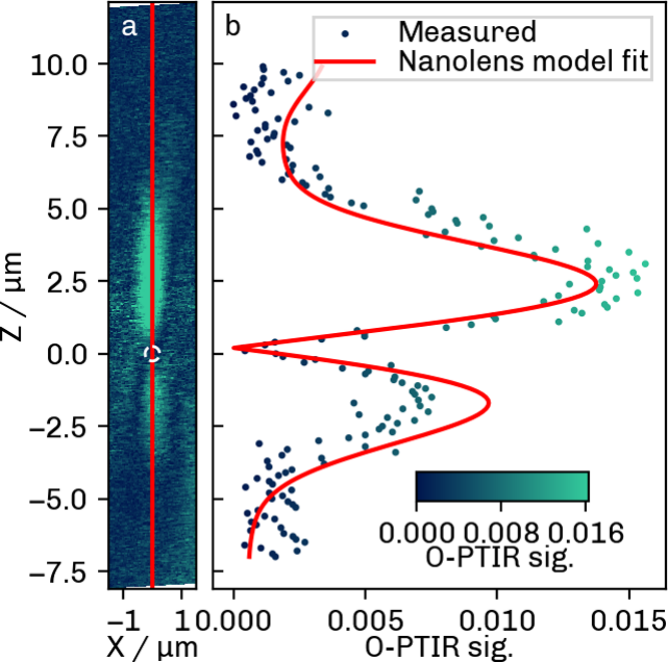
(a) O-PTIR z-scan at 1450 cm^–1^. The PSF was calculated along the mean of three lines (the central line in red). The white circle has the same radius as the bead and is placed at the approximate location of the bead. (b) Resulting PSF using the parameters of Table 1.

The corresponding theoretical PSF for the parameters of the O-PTIR instrument (Table 1) was found to correlate well with the experimental findings. As predicted by the theoretical model, the photothermal signal has two lobes, resulting from the change in focus position of the probe beam relative to the sample.

The fitting parameters included absorption cross section σ_*abs*_ and laser focus offset Δ*z*. The fitted absorption cross section of (2.7 ± 0.5)­10^–14^ m^2^ corresponds to the value reported by Pavlovetc et al.^19^ for 400 nm PS beads and is therefore lower than expected. This inconsistency is probably due to the fact that several experimental parameters had to be estimated with some uncertainty. Therefore, deviations from the true signal parameters could be expected. In particular, the beam waists were calculated from experimental fwhm values (see S-3, (S5)). Although the uncertainty of the fwhm measurement can be somewhat mitigated by averaging several measurements and images, it cannot be fully eliminated. One should also bear in mind that increasing the number of readings from which the average signal is obtained will also increase the pixel dwell time. The sample will then absorb more laser power, which may be detrimental to sample quality. Considering that the shape and relative height of the two signal lobes critically depend on the beam waists, the trade-off between the pixel dwell time and accuracy of measurement should be optimized in future research.

Further uncertainty arises from the approximate distance between the beam foci. It has been shown that the axial signal is not fully symmetric around the center of the spherical absorber and, in fact, the zero-crossing of the signal is shifted.^23^ While part of this shift is directly attributable to the finite size of the absorber and scales with the absorber radius, further shifting will be caused by aberrations when a high-NA objective is used. This asymmetry will affect the fit parameter of the focus offset Δ*z*. Furthermore, in the O-PTIR prototype presented here, the IR focus is kept at a fixed position. The VIS beam, on the other hand, is adjusted manually with 2 μm precision. Installing a motorized stage equipped with a piezo drive can lower this imprecision considerably and would ease quantification of the measurement uncertainty.

The axial offset Δ*z* between the pump and probe lasers dictates the shape of the axial signal and can be chosen to suppress either signal lobe, which is illustrated in Figure 10. The laser focus offset was increased to approximately 15 μm to suppress one of the lobes. After rotating the image by 4°, a Gaussian fit along the red line (b) yielded an axial resolution of 4.17 ± 0.34 μm. As predicted by theoretical calculations (Figure 5), the signal strength is much lower in the one-lobe case.

**Figure 10:**
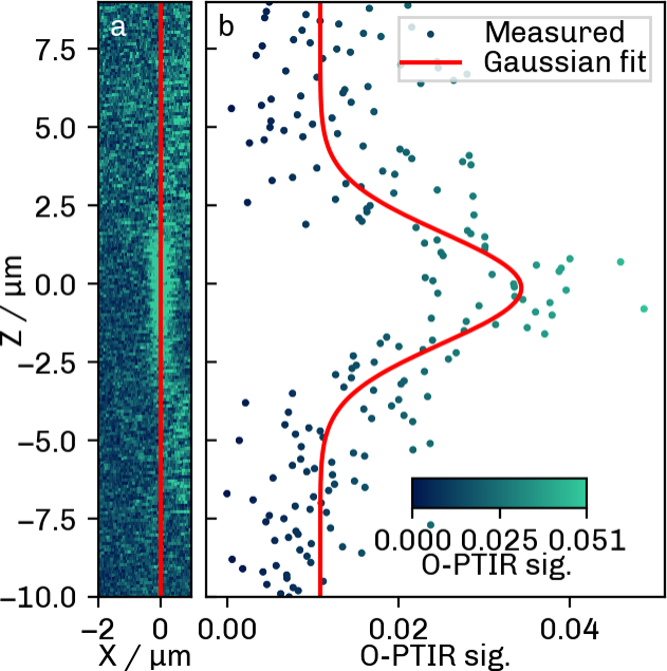
Axial O-PTIR PSF generated by an increase in laser focus offset to suppress one of the signal lobes. (a) *xz* image; (b) Gaussian fit along the red line in (a).

This result underscores the fact that the axial photothermal response does not follow the behavior of conventional optical imaging systems. Although the distance between the pump and probe beam foci can be set such as to minimize one of the lobes, the resulting response is not strictly Gaussian and resolution actually depends on the laser focus offset Δ*z*. On top of that, suppressing one lobe comes at the cost of the SNR and cannot be performed for larger particles because the required offset increases with the size of the particle, and eventually the signal is lost.

In addition, the axial transmission signal is not independent of the pump laser wavelength. As evident from ([Disp-formula eq12]), the induced refractive index change Δ*n*, that is, the thermal lens, increases with the Rayleigh range *z*_*R*,*h*_ of the pump laser and with the (wavelength-dependent) absorption cross section σ_abs_ of the particle.

While some of these issues can be solved by calibration, the two-lobed axial response is an intrinsic characteristic of photothermal detection. Other mechanisms like fluorescence-detected photothermal imaging^43^ and photoacoustic methods^44^ could be employed for signal read-out but will come at the drawback of requiring a fluorescing sample and low lateral resolution, respectively. To better understand the axial resolution in photothermal transmission microscopes, further studies should evaluate how the signal behaves for two closely spaced objects of various sizes.

## Summary

As expected, the commercial Raman and custom O-PTIR instruments exhibit similar PSF profiles in the radial direction despite the differences in the detection scheme (reflection vs transmission). For the O-PTIR setup, the spatial resolution was found to be approximately 653 nm in the *x* and 668 nm in the *y* direction, whereas the Raman instrument reached a resolution of 738 nm in the *x* and 840 nm in the *y* direction. The fact that spatial resolution is not fully axisymmetric in the *xy* plane is not surprising and can be explained by axial alignment errors such as sample tilt, which was stronger in the Raman measurements, and aberrations stemming from the optical elements.

Although bead diameters of the order of the probe wavelength were used, the experimental axial signal is in line with the nanolens diffraction model.^23,24^ Stronger discrepancies are expected for larger beads since the lower limit of the diffraction integral (eq 14) was approximated by 0 instead of the finite bead radius to obtain an analytical solution to the diffraction problem. For much larger objects of several micrometers, the point source model will no longer be valid due to reflections and standing waves inside the spherical absorber.^45^ Still, the nanolens model provides valuable insight into the axial signal response.

Theoretical calculations suggest that the photothermal signal amplitude is linear in the absorption cross section. This means that absorption cross sections can be recovered from the relative intensity distribution if the experimental parameters are known.

Photothermal *z*-scans performed at various axial offsets between the probe and the pump laser focus evince the two-lobed nature of the photothermal signal. By choosing an appropriate (large) focus offset, one can efficiently suppress either signal lobe to mimic the point-spread function of conventional imaging systems. Yet, this comes at the cost of weak signal intensity and low SNR. In general, the photothermal response strongly varies with the offset between the foci of the pump and the probe laser and cannot be described by a simple Gaussian function.

Against this background, axial photothermal scans in transmission mode are crucial for the alignment of the pump and probe beams to avoid zero-signal responses. Photothermal microscopes should therefore allow the user to control the parameter of the relative focus offset between the two lasers. Moreover, “autofocus” systems that translate the objective by a set amount to place the sample in the focus of the visible beam are likely insufficient.

Due to the dispersion-like (i.e., two-lobed) behavior of the photothermal signal around objects whose size is of the order of the probe-beam wavelength, z-stack images, when used to determine the vertical distribution of absorbers, are intrinsically prone to artifacts and should be interpreted with caution.

Photothermal image artifacts cannot be fully eliminated with the present state of knowledge. Where possible, one of the signal lobes should be suppressed to avoid mirror image artifacts. As lobe suppression has a detrimental effect on the SNR, a trade-off between signal strength and imaging fidelity must be made.

To assess the consequences of the dual-lobe signal on image quality, further study is needed to investigate the characteristics of the photothermal signal in micrometer-sized samples and nonspherical geometries.

## Supplementary Material



## Data Availability

The data underlying this study are openly available in Zenodo at DOI: 10.5281/zenodo.15190267.

## Notes

Theoretical considerations on the induced refractive index change and the axial photothermal signal as well as figures for the coordinate system used to calculate the electric field after refraction by a spherical particle (Figures S1, S2) and the O-PTIR xy image and PSF of a 500 nm bead at 1490 cm^–1^ (Figure S3). (PDF)
