# Supplementary material for: Not Just Better Resolution: A Detailed Study of the Signal Distribution in Mid-Infrared Optical Photothermal Imaging
Source: Anal Chem. 2025 Sep 22;97(39):21418–27. doi: 10.1021/acs.analchem.5c03194 (PMC12509188; doi:10.1021/acs.analchem.5c03194)
Supplement: Supplementary file 1 [file ac5c03194_si_001.pdf]

Supporting Information: Not Just Better Resolution: A Detailed Study of the Signal Distribution in Mid-Infrared Optical Photothermal Imaging

Elisabeth Holub,† Nikolaus Hondl,† Sebastian Wöhrer,† Bernhard Lendl,† and Georg Ramer*,†,‡

†*Institute of Chemical Technologies and Analytics, TU Wien, 1060 Wien, Austria*

‡*Christian Doppler Laboratory for Advanced Mid-Infrared Laser Spectroscopy in (Bio-)process Analytics, TU Wien, 1060 Wien, Austria*

E-mail: georg.ramer@tuwien.ac.at

Contents

1 Theoretical considerations **S-2**

1.1 Refractive index change S-2

1.2 Relation between peak power and average power S-2

1.3 Relation between beam waist and fwhm value S-3

1.4 Axial photothermal signal S-3

2 XY O-PTIR image at 1490 cm^{-1} **S-5**

References **S-5**

1 Theoretical considerations

1.1 Refractive index change

The refractive index response to a steady-state temperature profile is[S1]

$$\Delta n = \frac{dn}{dT}\frac{P_{diss}}{4\pi\kappa R}, \tag{S1}$$

where $\frac{dn}{dT}$ is the thermo-optic coefficient of the ambient medium,

κ is the thermal conductivity of the medium,

P_{diss} represents the average power absorbed and dissipated by the heat source (i.e. the particle).

With $P_{diss} = \sigma_{abs} I_{0,h}(z, \Delta z)$ and $I_{0,h}(z, \Delta z) = \frac{I_{0,h}}{1+\frac{(z-\Delta z)^2}{z_{R,h}^2}}$, the refractive index perturbation becomes,[S1 S2]

$$\Delta n(z, \Delta z) = \frac{\sigma_{abs} I_{0,h}}{4\pi\kappa R}\frac{dn}{dT}\left(1 + \frac{(z-\Delta z)^2}{z_{R,h}^2}\right)^{-1}, \tag{S2}$$

1.2 Relation between peak power and average power

In a simple model, the average power is the total power measured per unit time of the pulse period $\tau = t_1 + t_2$, while the peak power is the power emitted per unit time during period t_1 (Figure S1). Since the total energy emitted per pulse must be equal to the total energy emitted during the pulse period,

$$P_{max} \cdot t_1 = P_{avg} \cdot (t_1 + t_2) \Rightarrow P_{max} = P_{avg}\frac{t_1 + t_2}{t_1} = \frac{P_{avg}}{\beta}. \tag{S3}$$

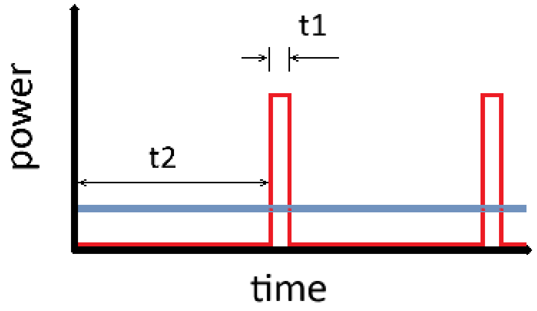

Figure S1: Power distribution of a pulsed laser. t_1 is the pulse width, and t_2 is the time during which the laser is not emitting. The average power is represented by the blue line.

1.3 Relation between beam waist and fwhm value

The intensity of a Gaussian beam is defined as[S3]

$$I = I_0 \cdot exp(-2\frac{r^2}{w^2}) \tag{S4}$$

The beam waist w i s then:

$$w = \sqrt{\frac{-2}{ln(\frac{I}{I0})}}r \tag{S5}$$

One can calculate the waist w if the width (r=fwhm/2) at the half maximum intensity(I = $I_0/2$) is known. The waist corresponds approximately to 0.85 times the fwhm value.

1.4 Axial photothermal signal

Starting from Equation 13,

$$\begin{aligned} E(r,z) = \frac{k}{iz} \exp\left(i\frac{kr^2}{2z} + ikz\right) \cdot \\ \int_R^\infty T(\rho)U_a(\rho) \exp\left(\frac{ik\rho^2}{2z}\right) J_0\left(\frac{k\rho r}{z}\right) \exp\left(-i\Delta\chi(\rho)\right)\rho d\rho, \end{aligned} \tag{S6}$$

we insert the Gaussian probing field distribution, which is given by:[S1,S4]

$$U_a(\rho) = E_0 \frac{\omega_0}{\omega(z)} \exp\left(-\frac{\rho^2}{\omega^2(z)}\right) \exp\left(-ikz - ik\frac{\rho^2}{2R_c(z)} + i\zeta_G(z)\right), \tag{S7}$$

where $\zeta_G(z) = \arctan \frac{z}{z_R}$is the Gouy phase and
$R_c = z\left[1 + \frac{z_R^2}{z^2}\right]$ is the local radius of curvature of the Gaussian (probe) beam.

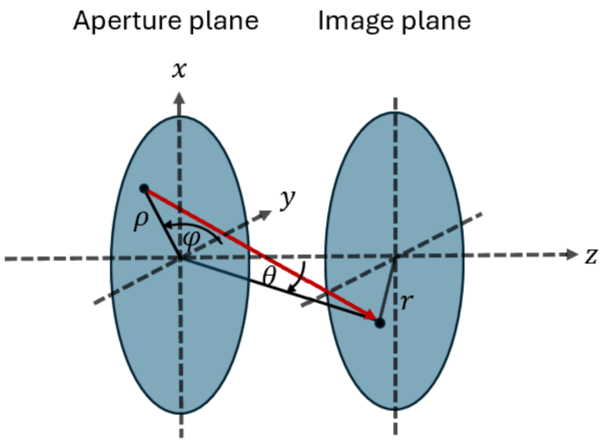

Figure S2: Coordinate system used to calculate the electric field after refraction by a spherical particle. ρ: radial coordinate of the aperture plane; r: radial coordinate in the image region.

To calculate the relative off-axis signal in the image plane , the radial coordinate r is transformed to an angle (Figure S2) via

$$\tan\theta = r/z. \tag{S8}$$

Exploiting the small-particle limit ($R \to 0$), the lower limit of integration can be set to 0.

Using the identities[S5,S6]

$$\left(\frac{2}{x}\right)^n \Gamma(n+1) J_n(x) = {}_0F_1(;, n+1, -\frac{x^2}{4}), \tag{S9}$$

$$\Gamma(a)_1F_1(a,b,z) = \int_0^\infty e^{-t}t^{a-1}\ {}_0F_1\ (:,b,zt)dt, \qquad Re(a) > 0 \tag{S10}$$

and[S7]

$${}_1F_1(a,b,z) = e^z\ {}_1F_1\ (b-a,b,-z), \tag{S11}$$

one can express the relative photothermal signal at a specific detection angle θ as

$$\Phi(\theta,z) = \exp\left(-\frac{k^2\tan^2\theta Re(\zeta^{-1})}{2}\right)\left(\exp\left(2R\Delta nk_0 \arg\left(\zeta\right)\right)\cdot \left|\Gamma(1+iR\Delta nk_0)\ {}_1F_1\ (-iR\Delta nk_0, 1, \frac{k^2\tan^2\theta}{4\zeta})\right|^2 - 1\right). \tag{S12}$$

2 XY O-PTIR image at 1490 cm^{-1}

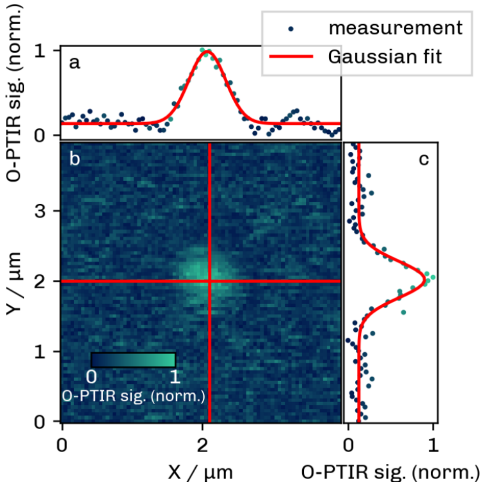

Figure S3: O-PTIR xy image at 1490 cm^{-1}(b) and resulting PSFs in x (a) and y (c) direction.

References

(S1) Selmke, M. Photothermal Single Particle Detection in Theory & Experiments. PhD thesis, Leipzig University, 2013; URN: urn:nbn:de:bsz:15-qucosa-126286.

(S2) Adhikari, S.; Spaeth, P.; Kar, A.; Baaske, M. D.; Khatua, S.; Orrit, M. Photothermal Microscopy: Imaging the Optical Absorption of Single Nanoparticles and Single Molecules. *ACS Nano* **2020**, *14*, 16414–16445, DOI: 10.1021/acsnano.0c07638.

(S3) Self, S. A. Focusing of spherical Gaussian beams. *Applied Optics* **1983**, *22*, 658, DOI: 10.1364/AO.22.000658.

(S4) Selmke, M.; Braun, M.; Cichos, F. Nano-lens diffraction around a single heated nano particle. *Optics Express* **2012**, *20*, 8055, DOI: 10.1364/OE.20.008055.

(S5) Johansson, F. Computing hypergeometric functions rigorously. *arXiv preprint arXiv:1606.06977* **2016**, DOI: 10.48550/ARXIV.1606.06977.

(S6) Kummer confluent hypergeometric function 1F1: Integral representations (formula 07.20.07.0002). https://functions.wolfram.com/07.20.07.0002.01.

(S7) Abramowitz, M.; Stegun, I. A. *Handbook of mathematical functions: with formulas, graphs and mathematical tables [conference under the auspices of the National science foundation and the Massachussetts institute of technology]*, unabridged, unaltered and corr. republ. of the 1964 ed ed.; Dover books on advanced mathematics; Dover publ: New York, 1972.
